# Invited discussant comments during the UCL-Penn Global COVID Study webinar ‘Alone Together: Loneliness Research and Social Health Innovation in Lockdown and Beyond’

**DOI:** 10.14324/111.444/ucloe.100004

**Published:** 2022-11-03

**Authors:** Kasley Killam

**Affiliations:** 1 Founder and Executive Director, Social Health Labs, www.socialhealthlabs.com

**Keywords:** social isolation, loneliness, social health, social support, resilience, innovation, social ecological model, emergency preparedness, Covid-19, pandemic

## Abstract

The coronavirus (Covid-19) pandemic has influenced people’s social relationships around the world in surprising ways. It has also underscored the importance of and accelerated innovation in solutions for social isolation and loneliness. This commentary offers takeaways from emerging research findings and a wide lens on the societal movement underway to create more socially connected communities.

**About the study**

The UCL–Penn Global COVID Study^1^ launched in April 2020 was a 12-month longitudinal study of the impact of Covid-19 on social trust, mental health and physical health. In collaboration with six institutions from Italy, Singapore, the United States, China and the United Kingdom,^2^ the study looks at the short- and longer-term effects of Covid-19 on individuals’ mental health and social relationships with others. Survey data were collected at three time-points: 17 April–14 July 2020 (Wave 1), 17 October–31 January 2021 (Wave 2) and 17 April–31 July 2021 (Wave 3).

**About the webinar**

Held online between 2 June and 28 July 2021, the study group presented research data at five online webinars, as part of the UCL Global Engagement Fund sponsorship, to discuss the lessons learned and invited policy makers and other subject experts to speak on the policy relevance and implications of the study findings. The recorded comments from these discussions focusing on the policy relevance and implications of each academic article were recorded as discussant articles and are published in this journal to be read alongside the research article being discussed.

These discussant articles are reviewed by members of the Editorial Board before being published. It is hoped that these discussant articles, read alongside the academic articles, will provide more holistic understanding of the issues at hand, how findings may inform policies in the coming months and/or assist in future crisis management strategies and aid decision-making, in an open and transparent manner.

The study was pre-registered https://osf.io/4nj3g/ on 17 May 2021) and ethical approval was obtained from the IOE (Institute of Education), UCL’s Faculty of Education and Society (University College London, UK) Ethics and Review Committee on 8 April 2020 (REC 1331).^1^

**Linked research article**

The linked research article to this discussion article cited here has been published in *UCL Open: Environment* following open peer review and made freely available to read as an open access article. Additionally, all previous versions and peer review reports are freely available to read as open access preprint articles from the journal’s preprint server by following the below DOI link and navigating to the version history of the published research article. Readers can find more information about how peer review works in the journal at ucl.scienceopen.com.

Carollo A, Bizzego A, Gabrieli G, Wong KK, Raine A, Esposito G. Self-perceived loneliness and depression during the Covid-19 pandemic: a two-wave replication study. *UCL Open: Environment*. 2022;(4):20. Available from: https://doi.org/10.14324/111.444/ucloe.000051

**Recorded webinar**

This discussion article comments on the findings presented during the following webinar that has been recorded and made freely available to readers to watch on-demand.

Summer Webinar 1 – Loneliness #GlobalCOVIDStudy. Available at: https://www.youtube.com/watch?v=WD1F9METk8A.

## Introduction

This commentary is based on a presentation titled ‘Alone But Not Lonely: Insights from the Pandemic’ that the author gave during the 2 June 2021 webinar on ‘Lockdown Duration Impacts Mental Health: Greece and UK’. Both the presentation and this commentary are in response to research by Carollo and colleagues and are intended to complement their findings with a broader view of pandemic trends and innovation in solutions for social isolation and loneliness. This commentary was prepared in August 2021 and reflects information that was current at that time.

## Discussant comments

Years from now, when future generations look back on the coronavirus (Covid-19) pandemic, what meaning will they make of it? What possible good can come from this terrible collective experience? Although the world is still in the throes of the pandemic, at least one key learning has already emerged: Social support is an essential source of resilience, one that we must invest in to prepare for future collective emergencies – not to mention benefit our day-to-day health and well-being.

As you will read in Carollo and colleagues’ article, the impact of lockdowns on people’s social relationships has not been as obvious as social scientists like me predicted. Indeed, in an article [1] for *Scientific American* published the week the United States (US) announced shutdowns, I warned that we might have to contend with more than one public health crisis. It stood to reason that prolonged social isolation due to quarantines and physical distancing requirements would trigger deep feelings of disconnection.

For many, this prediction has rung true. For instance, a recent national survey [2] of Canadians found that 61% feel somewhat or much lonelier as a result of the pandemic. In the US, three-quarters [3] of adults say the pandemic has made it more difficult for them to connect with friends. And Japan appointed a Minister of Loneliness in February 2021 to address rising suicide rates.

But as Carollo and colleagues’ findings and a growing body of research show, that conclusion is only part of the full picture. Consider a study [4] in *American Psychologist* that surveyed over 1500 people in the US three times before and during lockdown. It found no significant changes in loneliness and, surprisingly, an increase in perceived social and emotional support. Another study [5] out of the University of British Columbia and the University of California at Riverside tracked people in the US, the United Kingdom (UK), and 26 other countries starting before the pandemic. The participants reported feeling equally as socially connected during lockdown and, in fact, somewhat less lonely than before. Similarly, when researchers surveyed [6] 40,000 people aged 13–40 in countries around the world, 67% said their friendships were not impacted by the pandemic – and among the remaining people, 47% actually felt closer to their friends than before^3^.

## Early research insights

As the pandemic continues and more research is published, we will be better able to explain these findings and tease out the nuances of people’s social experiences during the pandemic. But already several important takeaways are clear, and together they offer a hopeful message. The first lesson is that social isolation, which is objective, does not necessarily cause loneliness, which is subjective. People can – and indeed many did – feel emotionally connected to others despite being physically separated.

Another lesson is that social support is an essential source of resilience. For instance, the World Happiness Report [7], published in March 2021, identified the quality of people’s relationships, the size of their social networks, their sense of connectedness and resonance with others, and their prosocial behaviours as key drivers of psychological well-being during the pandemic. In addition to protecting mental health, social support protects people’s physical health. For instance, one study [8] found that socially cohesive neighbourhoods had lower infection rates and fewer deaths from Covid-19.

There are a few possible explanations for this. One is practical: if you know, like and trust people in your community, you are more likely to exchange useful health information with each other, such as where to buy masks or how to schedule a vaccination appointment, and to help each other out, such as by dropping off groceries for an elder or immunocompromised neighbour. Another explanation could be psychological: when you know, like and trust people in your community, you are more likely to feel a sense of responsibility for each other and support one another emotionally, which in itself can confer health benefits.

As researchers have observed, and many of us have experienced firsthand, we feel connected from engaging in individual interactions and relationships with romantic partners, friends, family members, neighbours, and co-workers. A diverse network of social ties can even protect people from heart disease [9] and mortality [10]. But we also feel connected from a broader sense of community and camaraderie. This was evident in the use of mutual aid groups during the pandemic. Mutual aid groups are voluntary, grassroots networks of community members who self-organise to match services and resources to the people who need them, such as picking up people’s prescriptions or making friendly phone calls to check in. Evidence [11] suggests that these efforts helped not only the receivers, but also the givers.

## Innovation in social health

These insights contribute to a broader shift in our understanding of health as not only physical and mental, but also social. I define *social health* as the dimension of well-being that comes from connection and community^4^. Growing awareness about the importance of social health and motivation to alleviate widespread loneliness are fuelling a wave of innovation across sectors. Ongoing efforts in government, healthcare, technology and urban design offer examples.

In government, multiple countries have recognised the public health threat of widespread social isolation and loneliness and therefore are investing in solutions. The value of these government efforts is in validating the issue and allocating resources to address it. For instance, both the UK and Japan, as mentioned, have appointed Ministers of Loneliness to coordinate national strategies for more socially connected communities. In the US, numerous policymakers have proposed legislation [12] to allocate federal spending toward local solutions for social isolation and loneliness.

In healthcare, the practice of social prescribing is gaining traction, wherein doctors ‘prescribe’ social engagement for isolated or lonely patients and link them to community resources. A study [13] in the *British Medical Journal* found that patients who received social prescriptions felt more socially connected and less lonely – and therefore decreased their use of primary care services. Similarly, the number of hospital admissions was 21% lower [14] among participants in a US-based social prescribing programme.

In technology, an increasing number of startups [15] are designing apps and digital platforms to facilitate more meaningful connection than traditional social media. The goals of these tools include making new friends, staying in touch with loved ones, having deeper conversations, finding communities and events online or in person and bridging interpersonal or intergroup divides.

In urban design, research has shown that the way cities, neighbourhoods, buildings and the other physical spaces that we live, work and play in can either help or hinder our social relationships^5^. As a result, more developers, architects and urban planners are incorporating these insights into their designs. During the pandemic, numerous organisations published reports with recommendations on this topic, including Healthy Places by Design [16], HKS Architects [17], and the Future Spaces Foundation [18].

More broadly, the global dialogue about social isolation and loneliness has taken off. At Social Health Labs, we lead a thought leadership series^6^ with the Foundation for Social Connection to generate cross-sector solutions^7^. The Togetherness Hub^8^ brings together innovators in this space from around the world to connect and share virtually. And organisations from numerous countries – including the US Coalition to End Social Isolation and Loneliness, the UK Campaign to End Loneliness, and Australia’s Ending Loneliness Together – have formed the Global Initiative on Loneliness and Connection^9^ to collaborate and advance solutions. These efforts mark just the beginning of the social health movement.

## A path forward

Now is the time to build on this unprecedented momentum and incorporate the learnings from Covid-19 research into longer-term strategies. For one, communities around the world should proactively strengthen social health to build resilience as part of their emergency preparedness strategy. Reinforcing existing mutual aid networks is a good first step. But city governments and neighbourhood associations could also take an active role in planning, coordinating and funding other approaches to strengthen social support systems that are tailored to local needs and preferences^10^.

More generally, we must make social health a priority in society. At a minimum, the digital divide and other inequities that became apparent during the pandemic need to be addressed so that people have the resources they need – including internet access and technology skills – to stay connected remotely. Moreover, we must continue to innovate in different sectors at local, regional, national and global levels to alleviate social isolation and loneliness and improve people’s social well-being.

One way to guide this innovation is by mapping current efforts and identifying gaps using the social-ecological model. Commonly used by public health practitioners, this framework contextualises a given person’s health in relation to interpersonal, community, institutional and societal influences. It also points to opportunities for intervention and impact from upstream (e.g., systemic) to downstream (e.g., individual) levels^11^.

Years from now, when future generations look back on the Covid-19 pandemic, I hope that they will applaud us for incorporating learnings from this tragedy and taking action to strengthen our relationships and communities. But it is up to each of us to make that a reality. As you read Carollo and colleagues’ paper and the other articles in this special edition, I invite you to consider how you can make the most of this turning point and build up social health as a driver of resilience and well-being in your life, community and work.

## Notes

1 Wong KK, Raine A. Covid-19: Global study of social trust and mental health. Available from: https://globalcovidstudy.com [Accessed: 11 November 2021].

2 Wong KK, Raine A. Covid19: Global social trust and mental health study. 2020. https://doi.org/10.17605/OSF.IO/FE8Q7.

3 For an updated account of research on loneliness prevalence published in 2022, please see: https://www.psychologytoday.com/us/blog/social-health/202204/did-people-get-lonelier-during-the-pandemic

4
https://www.socialhealthlabs.com/social-health

5 For an updated account of research on urban design and social capital published in 2022, please see Chapter 8: https://islandpress.org/books/making-healthy-places-second-edition

6 See: https://www.socialhealthlabs.com/connect

7 For a white paper with recommendations from this series published in 2022, please see: https://www.socialhealthlabs.com/connect-report

8 See: https://www.togethernesshub.com/community/

9 See: https://www.gilc.global/

10 For additional recommendations related to emergency preparedness published in 2022, please see: https://generations.asaging.org/improving-social-health-community-resilience

11 For additional recommendations related to this framework published in 2022, please see: https://www.socialhealthlabs.com/connect-report
